# Variations in the relative age effect with age and sex, and over time—Elite-level data from international soccer world cups

**DOI:** 10.1371/journal.pone.0264813

**Published:** 2022-04-28

**Authors:** Arve Vorland Pedersen, Tore Kristian Aune, Terje Dalen, Håvard Lorås

**Affiliations:** 1 Department of Neuromedicine and Movement Science, Faculty of Medicine and Health Sciences, NTNU, Trondheim, Norway; 2 Department of Sports Science, Faculty of Education and Arts, Nord University, Levanger, Norway; 3 Department of Teacher Education, Faculty of Social and Educational Sciences, NTNU, Trondheim, Norway; Universidade Federal de Mato Grosso do Sul, BRAZIL

## Abstract

The relative age effect (RAE) is a statistical bias observed across sport contexts and consists of a systematic skewness in birth date distribution within an annual-age cohort. In soccer, January 1^st^ is the common cut-off date when categorizing players in competitions according to their chronological age, which potentially disadvantages those within the cohort who were born later in the year. Thus, relatively older soccer players in their cohort can be favored in talent identification, selection, and development. The aim of the current study was to investigate the variations in RAE in male and female international youth world-cup tournaments (U17 and U20) in the period from 1997–2019 and in international senior world-cup-tournaments from 2006–2019. A total of 20,401 soccer players participating in 47 different tournaments were analyzed. The birthdate distributions were categorized into four quartiles (January-March, Q1; April-June, Q2; July-September, Q3; October-December, Q4) and compared to a uniform distribution using Chi-square analysis with Cramer’s V (*Vc*) as a measure of effect size. Based on the existing data concerning RAE in elite junior and senior soccer, it was hypothesized that: (I) the RAE is present in youth soccer world cup tournaments but is stronger in male players than in female players; (II) the younger the soccer players, the stronger the RAE; and (III) the RAE in world cup soccer tournaments has strengthened over time. All these hypotheses were supported by the data; novel findings included that the effect has now entered women’s soccer, and in men’s soccer it persists into senior world cup tournaments. Thus, a strong RAE bias occurs in selection among elite soccer players competing in international world cup tournaments.

## Introduction

The relative age effect (RAE) is well-known after being thoroughly researched over the years. The RAE is perhaps best described as the systematically skewed birth date distribution within an age cohort, thus disadvantaging those in the cohort who are born relatively later in the year [1]. The effect has been reported in school systems [2–4], within certain medical diagnoses [5, 6], in cognitive tasks [7], and in sports [8–11].

Grondin, Deshaies, & Nault [12] first observed the RAE in sports—for hockey but not for volleyball (both of which were investigated in the same study)—but the discovery of the effect is most often credited to Barnsley, Thompson & Barnsley [13], who reported it in Canadian ice hockey. Since then, the RAE has been identified within a number of sports, including basketball [14, 15], tennis [16, 17], alpine skiing [18, 19], handball [20, 21], and soccer [22, 23].

Individuals born early in a selection year will typically be more physically developed compared to their counterparts born late in the year, as has been shown by Bliss & Brickley [24], Dalen et al. [25], and Hirose [26], among others. Another explanation of the superior performances of relatively early-born children is that they have up to a year more experience within their sport, an effect which was termed the “initial performance advantage” by Helsen et al. (p. 630) [27]. When selected, players will also benefit from effects like the ‘Pygmalion effect’ [28]—which describes how individuals’ achievements are products of the expectations placed upon them—and the ‘Matthew effect’ [29], which describes the effect of accumulated advantage, often stated as “the rich get richer, and the poor get poorer” ([30]). Furthermore, unevenly distributed facilities favoring selected individuals will enhance the RAE, most notably coaches, training facilities, and the like. Consequences of the RAE may include favoring the physically precocious at the expense of real talent, which is a waste of potential [31, 32], as well as dropout from the sport [33–35].

In sports where the physical characteristics of athletes are less important, the RAE seems to be less prominent or absent altogether [36, 37]. Furthermore, within some sports where it is advantageous to be of smaller stature, selection favors the relatively later born; notable examples are dance [38] or horse racing [39], where the effect seems to work in the opposite direction and relatively late-born athletes are in the majority (an effect that has been termed the RAE reversal phenomenon [37, 40–41]. However, this reversal is the same RAE mechanism. A real inverse effect has been shown in a few studies of athletes at the absolute top level [42–44]. These results may suggest that those who are able to survive in an environment with strong RAE may subsequently benefit from the extra competition and the extra effort they put in to overcome the effect [45].

Research has shown variability of the RAE in women’s sports, probably dependent on several interacting constraints [10]. In sports with fewer participants the competition is less fierce, and selection starts later, and as a consequence it weakens the RAE [1]. In some sports there are fewer female athletes, which can be hypothesized to have an impact on the RAE. Furthermore, physical differences are somewhat smaller among girls than among boys [46, 47]. In addition, girls reach puberty earlier than boys, who reach their peak height velocity nearly two years later [48]; as a result, the largest differences coincide less with the timing of the strictest selection regimes. While a meta-review reported that the RAE is rarely found among female athletes [10], the RAE was indeed found in all age groups of French female soccer players between the ages of 8 and 17 years [49], and across several female youth sports during the 2012 Winter Youth Olympic Games [50].

Soccer is one of the sports where the RAE is observed at every level, from youth players up to the senior level, from recreational to national, international, and the absolute elite level—the FIFA World Cup. This comes as no surprise, given that soccer is the world’s largest sport and has an increasingly high degree of competition and increasingly early selection [51]. Furthermore, soccer is a sport in which players benefit from being physically precocious [52].

Since Barnsley et al. [53] and Dudink [54] first reported the RAE in soccer, albeit in a time when cut-off dates were more variable, and also different from today, it has been consistently shown that the RAE is strong and pervasive within male youth soccer (see reviews by Sierra-Diaz et al. [55] and de la Rubia et al. [11]). The general trends are that the effect is stronger among the younger players and is gradually waning. Furthermore, the RAE seems to have grown stronger over time. In female soccer, reports have been scarcer, and the RAE is generally weaker. However, the same general trends are evident, in particular when considering the most recent studies [56–58]. In male international soccer, there is a strong and pervasive RAE in U17 World cup tournaments [59, 60]. Takacs and Romann [61] found medium-to-strong effects in the UEFA Youth League, whereas Yagüe et al. [62] found RAEs, though mostly small, in all top ten ranked European senior leagues, apart from the Belgian. Of course, each of those leagues included many (even mostly) players who were not international level players; also, it should be noted that the RAE in adults is indirect (a carry-over effect of the RAE). However, the finding indicates that relatively early-born players are over-represented among youth team players in the big clubs, as well as among senior teams in the big leagues. Among female international players, a quite recent study reported small, insignificant effects in Olympic tournaments since 1996 [63]. Sedano et al. [57] had previously reported a clear effect among Spanish national teams; however, their sample was combined from U17, U19, U21, and senior players, with a rather small total N of 232. Götze and Hoppe [22] did not find RAEs among German female national team players (U19, U20, and senior); however their samples were even smaller.

In soccer, as in sports in general, the competition is growing increasingly fierce, and more and more players invest more and more time, especially as salaries and transfer values are increasing almost exponentially [64–66]. Furthermore, Elferink-Gemser et al. [51] also reported a trend of increasing physical demand in soccer. Thus, the RAE could be expected to become stronger over time. In professional soccer players from ten European countries, Helsen et al. [67] showed that over a 10-year period from the 2000–2001 to the 2010–2011 competitive seasons, clear and persistent RAEs could be found. So far, data for WC tournaments have been too scarce for such longitudinal comparisons, apart from the above-mentioned datasets reporting results from a few tournaments.

The aim of the present study was to, more directly, compare the existence or not, as well as the strength of the RAE across sex and age, and over time. To that end, comparable data were needed, and thus the players should be performing at similar levels, and under similar rules. Such groups were found among players participating in FIFA’s World Championship tournaments. Players across all groups would be performing at the highest possible level for their age group, and the selection process is similar across groups. Hence, the variations within the RAE could be studied with less bias, and less uncertainty. The present data include the male U17s (even though these have been frequently reported, and Steingröver et al. [60] exhausted the results up until 2017) as well as the female U17s (who have not been studied previously). Furthermore, data from the somewhat less reported male U20s are included, together with the female U20s. For comparison, the four most recent senior WCs for men and for women were included. This way, variations of the RAE could be studied and compared across age and sex, and over time (chronologically across tournaments). The following hypotheses were tested: 1) The RAE is present in youth soccer world cup tournaments, but stronger in male compared to female players; 2) The younger the players, the stronger the RAE; and 3) The RAE has grown stronger over time.

## Materials and methods

### Samples

Players’ birthdates were obtained from the official Fédération Internationale de Football Association (FIFA) websites [68]. The Under-17 (U-17) Soccer World Championships take place every second year; thus, data from 12 tournaments for male players and 6 tournaments for female players were available from the period of 1997–2019. Similarly, the Under-20 (U-20) Soccer World Championships are held every second year, resulting in a total of 12 tournaments for male players and 9 tournaments for female players from 1997–2019. For comparison, the birthdates of players participating in the four most recent female and male senior soccer WC tournaments were also obtained from the FIFA websites. The total sample comprised 20,401 soccer players participating in 47 different tournaments representing a total of 104 countries all over the world, as teams (countries) from all continental member associations were represented in each tournament (see Table 1 for overview). In addition to players’ birth dates, information was obtained on sex and tournament.

**Table 1:** Main characteristics of the total sample.

Female players						Male players					
Tournaments						Tournaments					
U17	*n*	U20	*n*	Senior	*n*	U17	*n*	U20	*n*	Senior	*n*
2008	336	2002	219	2007	335	1997	288	1997	432	2006	734
2010	336	2004	251	2011	336	1999	288	1999	431	2010	736
2012	336	2006	336	2015	552	2001	290	2001	437	2014	736
2014	336	2008	335	2019	552	2003	320	2003	480	2018	736
2016	336	2010	336			2005	320	2005	505		
2018	336	2012	336			2007	507	2007	503		
		2014	336			2009	504	2009	504		
		2016	337			2011	502	2011	504		
		2018	336			2013	503	2013	504		
						2015	504	2015	504		
						2017	504	2017	504		
						2019	504	2019	504		

### Procedures

The process of collecting data from the FIFA website consisted of locating world cups registered under tournaments, in which each tournament has their own site. Next, under each team, the player information is listed. FIFA has chosen January 1^st^ as the cut-off date in junior tournament regulations. For within-year effects (i.e., typical relative age effects), birth dates were coded into four quartiles (Q1: January-March, Q2: April-June, Q3: July-September, Q4: October-December). The reason for including so many tournaments, outside increasing the total *n* of included players, was to investigate whether RAE would vary in any way over time. The study excluded tournaments before 1997, which was when FIFA introduced January 1^st^ as the cut-off date for players participating in youth WC tournaments [68]. Thus, players who participated in the male U17 tournament in 1997 would have been born in 1980 or later and were 26 years or younger in 2006—the year of the first senior world cup tournament included in the present study. These players would have been 30 years old in 2010, 34 years in 2014, and 38 in 2018 (the other senior tournaments included in this study). Fewer and fewer players participating in the WC in 2014–2018 would have been selected under regulations setting the cut-off date on a date other than Jan 1^st^, and the total number of such players would be relatively small. For female players, every youth tournament was held after the Jan 1^st^ cut-off had been imposed, and the relative numbers would be similar to those for men in the senior tournaments.

Some countries apply different cut-off dates in domestic competitions, which might have affected the selection of their national teams. (For example, England uses August 1^st^ as a cut-off.) Whatever the effect of such variations in cut-offs, it would work against the RAE as it is usually defined (i.e., by calendar year). The English players benefiting the most from the RAE in the national youth system would be those born in the third and fourth quartiles, thus belonging to the pool of later-born players in the present data. Had these birth dates been re-coded, the RAE might be even stronger. Given the sample size of the total dataset (20,401), these effects should be relatively small considering the large pool of data. Furthermore, given that FIFA and the 6 other international football confederations all apply the same cut-off date (1^st^ January), nearly all national associations also apply the same rule. The relatively few nations applying other cut-off rules therefore make up fewer than 5% of the total sample of players.

Quite a lot of players were one or two years younger than the oldest in their age group (e.g., 16-, and 15-year-olds playing in the U17 WC). These were, however, not differently distributed than the oldest cohort in the age group, and all players participating in the same WC-age group (U17, or U20) were thus pooled and analyzed together as one group.

Players may have been included in several tournaments due to having represented their country at several age levels, but they would rarely play twice in the same tournament (age group). In any case, such overlap is assumed to not systematically favor any quartile and would be of small effect within such a large dataset.

### Statistical analysis

In order to assess differences across the relative age quartiles, the observed distributions were analyzed by means of Chi-square tests (χ2) for each tournament. Due to the multinational sample in the current study, it was not possible to take into consideration the potential differences in birth rates per month that might exist across countries. Therefore, an equal distribution of births across all months and years was assumed for all analyses (see also [67]). Effect sizes for the chi-square tests were calculated with Cramer’s V (*Vc*), with strength of association interpreted as *low* = .1 to .3, *moderate* = .3 to .5, and *high* > .5 [69]. Potential associations among the RAE magnitudes (effect size) across time points (i.e., year of tournament) were analyzed with Spearman’s rho (*ϕ*). The strength of associations for Spearman’s rho (*ϕ*) was interpreted as *small* = .2, *moderate* = .5, and *strong* = .8) [69]. The statistical analyses were performed in SPSS (Version 25.0, IBM, US), and *p* < .05 was used as the threshold for statistical significance.

## Results

### Under-17 tournaments

**Fig 1:**
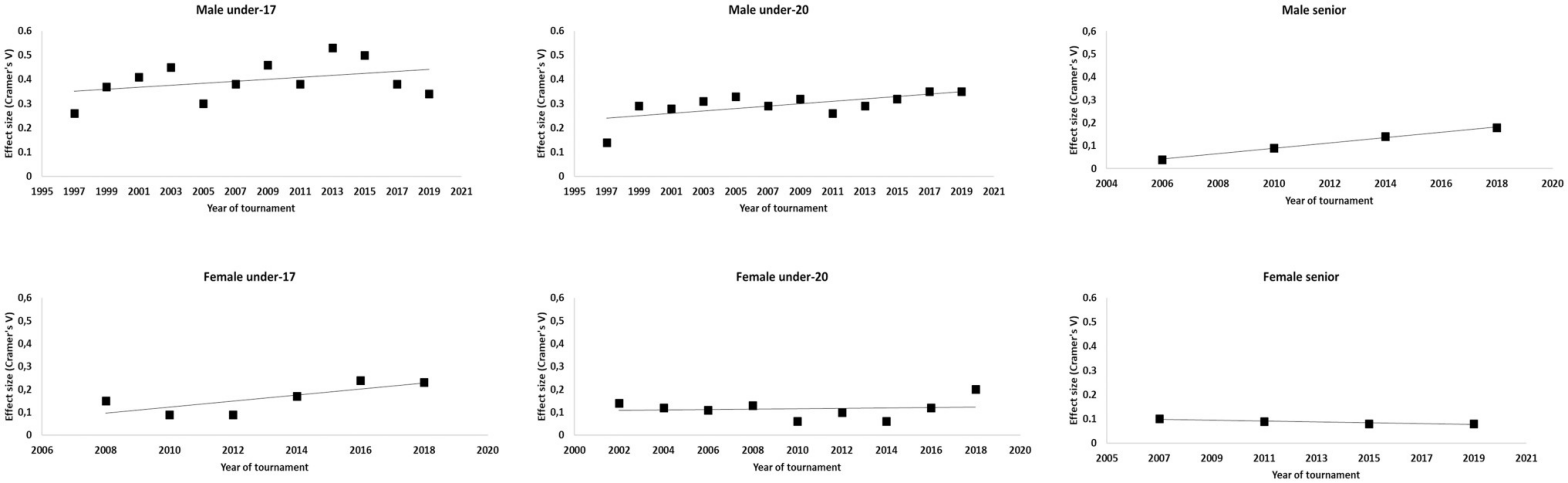
Scatterplots of effect sizes for RAE across gender, level and tournaments (top graphs for males, bottom graphs for females).

A significant RAE was evident in every one of the 12 male U17 tournaments (Table 2). Furthermore, as depicted in Fig 1, there were tournament-to-tournament differences in the relative magnitudes of the RAE. Evidence showed an increasing trend across WC tournaments, indicated by a significant linear relationship between RAE effect size and year of tournament from 1997 to 2015 (Spearman’s *ϕ* = .72, *p* = .018, *strong association*). In the two most recent tournaments, however, a small decrease in RAE effect size was observed (2017: *Vc* = .38 and 2019: *Vc* = .34). The strongest effect (*Vc* = .53) was found in the 2013 WC, in which as many as 46.3% of players were born in Q1 compared with 12.5% in Q4, and a total of 71.5% of players were born within the first six months of the year.

**Table 2:** Distribution of birth dates in *male* soccer players participating in the under-17 world cup from 1997–2019.

Quartile											
	Q1		Q2		Q3		Q4				
Year	*n*	*%*	*n*	*%*	*N*	*%*	*n*	*%*	χ^2^	*p*	*ES* ^1^
1997	104	36.1	56	19.4	68	23.6	60	20.8	20.01	< .001	0.26
1999	112	38.9	76	26.4	62	21.5	38	13.2	39.93	< .001	0.37
2001	118	40.7	78	26.9	54	18.6	40	13.8	48.24	< .001	0.41
2003	134	41.9	92	28.7	49	15.3	45	14.1	65.67	< .001	0.45
2005	115	35.9	90	28.1	57	17.8	58	18.1	29.22	< .001	0.30
2007	207	40.8	118	23.3	99	19.5	83	16.4	72.63	< .001	0.38
2009	219	43.5	128	25.5	81	16.1	76	15.1	104.63	< .001	0.46
2011	202	40.2	128	25.5	89	17.7	83	16.5	71.70	< .001	0.38
2013	233	46.3	127	25.2	80	15.9	63	12.5	137.71	< .001	0.53
2015	229	45.4	127	25.2	77	15.3	71	14.1	127.34	< .001	0.50
2017	202	40.1	123	24.4	109	21.6	70	13.9	73.17	< .001	0.38
2019	191	37.9	132	26.2	108	21.4	73	14.5	58.78	< .001	0.34

^1^ ES: Effect size (Cramer’s *Vc*)

In the female U17 championships (see Table 3), there was no significant RAE in tournaments from 2008 to 2012. However, the effect increased steadily from tournament to tournament and reached significance for the first time in the 2014 championship. Thereafter, it remained significant and of similar strength in the 2016 and 2018 championships. Due to such tournament-to-tournament differences, no significant association was found between year of tournament and RAE effect size (Spearman’s *ϕ* = .75, *p* = .084, *strong association*, see Fig 1). Also, the RAE effect sizes in female U17 world championships were lower than those in the corresponding male U17 world championships.

**Table 3:** Distribution of birth dates in *female* soccer players participating in under-17 world cup 2008–2018.

Quartile											
	Q1		Q2		Q3		Q4				
Year	*n*	*%*	*n*	*%*	*n*	*%*	*n*	*%*	χ^2^	*p*	*ES* ^1^
2008	95	28.3	91	27.1	88	26.2	62	18.5	7.90	.048	0.15
2010	95	28.3	86	25.6	75	22.3	80	23.8	2.63	.453	0.09
2012	96	28.6	75	22.3	86	25.6	79	23.5	3.03	.391	0.09
2014	105	31.3	88	26.2	72	21.4	71	21.1	9.24	.027	0.17
2016	107	31.8	101	30.1	63	18.8	65	19.3	19.33	< .001	0.24
2018	113	33.6	88	26.2	74	22.0	61	18.2	17.72	< .001	0.23

^1^ ES: Effect size (Cramer’s *Vc*)

### Under-20 tournaments

In the male U20 data (see Table 4), the pattern is similar to that in the U17 championships, with a significant RAE found in all tournaments. Although the effect sizes are smaller compared to those in U17 tournaments, the U20 male WCs also showed a significant increase (see Fig 1) in effect size over time (Spearman’s *ϕ* = .64, *p* = .024, *moderate association*). The largest effect (*Vc* = .35) in male U20 tournaments was found in 2019, a tournament in which 38.1% of players were born in Q1 compared to 14.5% in Q4, and a total of 64.3% of players were born in the first half of the year.

**Table 4:** Distribution of birth dates in *male* soccer players participating in under-20 world cup 1997–2019.

Quartile											
	Q1		Q2		Q3		Q4				
Year	*n*	*%*	*n*	*%*	*n*	*%*	*n*	*%*	χ^2^	*p*	*ES* ^1^
1997	133	30.8	106	24.5	98	22.0	95	22.0	8.32	.004	0.14
1999	161	37.4	95	22.0	97	22.5	78	18.1	37.10	< .001	0.29
2001	150	34.3	115	26.3	107	24.5	65	14.9	33.51	< .001	0.28
2003	173	36.0	129	26.9	107	22.3	71	14.8	45.54	< .001	0.31
2005	181	35.8	144	28.5	113	22.4	67	13.3	55.42	< .001	0.33
2007	173	34.4	149	29.6	98	19.5	83	16.5	42.73	< .001	0.29
2009	185	36.7	130	25.8	116	23.0	73	14.5	50.81	< .001	0.32
2011	176	34.9	132	26.2	104	20.6	92	18.3	33.14	< .001	0.26
2013	186	36.9	123	24.4	87	17.3	108	21.4	43.32	< .001	0.29
2015	185	36.7	136	27.0	105	20.8	78	15.5	50.24	< .001	0.32
2017	188	37.3	145	28.8	93	18.5	78	15.5	60.31	< .001	0.35
2019	192	38.1	132	26.2	107	21.2	73	14.5	60.03	< .001	0.35

^1^ ES: Effect size (Cramer’s *Vc*)

At the U20 level for female players (Table 5), a significant RAE first appeared in the 2018 tournament. Thus, there was no association between year of tournament and effect size (Spearman’s *ϕ* = .08, *p* = .83, see Fig 1).

**Table 5:** Distribution of birth dates in *female* soccer players participating in the under-20 world cup 2002–2018.

Quartile											
	Q1		Q2		Q3		Q4				
Year	*n*	*%*	*n*	*%*	*n*	*%*	*n*	*%*	χ^2^	*p*	*ES* ^1^
2002	65	29.7	55	25.1	56	25.6	43	19.6	4.55	.220	0.14
2004	69	27.5	71	28.3	54	21.5	57	22.7	3.53	.331	0.12
2006	94	28.0	84	25.0	89	26.5	69	20.5	4.22	.244	0.11
2008	86	25.7	89	26.6	94	28.1	66	19.7	5.42	.151	0.13
2010	83	24.7	83	24.7	92	27.4	78	23.2	1.21	.752	0.06
2012	83	24.7	89	26.5	93	27.7	71	21.1	3.32	.354	0.10
2014	79	23.5	81	24.1	83	24.7	93	27.7	1.44	.711	0.06
2016	98	29.1	89	26.4	74	22.0	76	22.6	4.60	.212	0.12
2018	106	31.5	93	27.7	63	18.8	74	22.0	13.24	< .001	0.20

^1^ ES: Effect size (Cramer’s *Vc*)

### Senior tournaments

The RAE was altogether absent in senior male players until the 2014 tournament. The RAE was also evident in the 2018 tournament, with a larger effect size compared to 2014 (see Table 6 and Fig 1). Regardless of significant effects in each tournament (possibly redundant anyway, according to Gibbs, Shafer, & Dufur, 2015), a linear and significant increase can be seen in RAE magnitude across the four most recent male senior tournaments (Spearman’s ϕ = .99, p < 0.01). No significant RAE was found in any of the four most recent senior female tournaments (Table 7 and Fig 1) and there was no significant association between year of tournament and effect size (Spearman’s ϕ ≤ .95, p = .051; see Fig 1).

**Table 6:** Distribution of birth dates in *male* soccer players participating in the senior world cup 2006–2018.

Quartile											
	Q1		Q2		Q3		Q4				
Year	*n*	*%*	*n*	*%*	*n*	*%*	*n*	*%*	χ^2^	*p*	*ES* ^1^
2006	189	25.7	182	24.8	190	25.9	173	23.6	1.13	.790	0.04
2010	208	28.3	192	26.1	163	22.1	173	23.5	6.64	.082	0.09
2014	220	29.9	190	25.8	180	24.5	146	19.8	15.23	< .001	0.14
2018	229	31.1	191	26.0	180	24.5	136	18.5	23.91	< .001	0.18

^1^ ES: Effect size (Cramer’s *Vc*)

**Table 7:** Distribution of birth dates in *female* soccer players participating in the senior world cup 2007–2019.

Quartile											
	Q1		Q2		Q3		Q4				
Year	*n*	*%*	*n*	*%*	*n*	*%*	*n*	*%*	χ^2^	*p*	*ES* ^1^
2007	87	26.0	94	28.1	83	24.8	71	21.2	3.32	.350	0.10
2011	89	26.5	92	27.4	83	24.7	72	21.4	2.84	.432	0.09
2015	150	27.2	148	26.8	130	23.6	124	22.5	3.72	.302	0.08
2019	134	24.3	155	28.1	124	22.5	139	25.2	3.91	.303	0.08

^1^ ES: Effect size (Cramer’s *Vc*)

## Discussion

The present study investigated variations of the RAE across age and sex as well as over time (chronologically across tournaments). Main findings indicated a significant and increasingly stronger RAE in male U17 and U20 tournaments, and a significant RAE in the two most recent male senior tournaments. Among female players, a significant RAE was only found in the two most recent U17 tournaments, and the most recent U20 tournament, whereas no significant RAE was found in the past four senior female tournaments. It was indeed hypothesized that the RAE would be found in male but not in female players (or at least to a much smaller extent). Furthermore, it was hypothesized that the effect would be stronger with younger players (U17 vs. U20 and senior). Finally, it was hypothesized that the effect would grow stronger over time, thus manifesting more clearly in the most recent tournaments. All these hypotheses were supported by the present data. The most surprising findings were that the effect has now entered women’s soccer and that the effect in men’s soccer is so strong that a carryover RAE is evident in the two, most recent male, senior WC tournaments.

Some of the results should be familiar to the reader, as they have been presented by others previously—most notably, the effects in the U17 tournaments [59, 60] and the RAE in the male senior WC in 2014 [60]. However, the present study attempted to portray the overall picture of the RAE at the highest level; thus, it presents comparable data for all age groups (U17, U20, and senior), for both male and female players (never done prior to this study), and over an extended time period.

The present finding that the RAE is strongest among the youngest players is not surprising considering that this is the period with the largest variations in physique and anthropometry due to age [46–48]. Furthermore, for boys, this period coincides with the most intense period of selection and talent scouting [70]. In the senior male WC, no RAE was evident until the 2014 tournament (as was previously shown by Steingröver et al. [60]), and it persisted in 2018. The observed RAE in senior male soccer in the two most recent WC-tournaments in the present study stands in contrast to several findings of reversal RAE in senior male team sports (for review, see de la Rubia et al. [11]).

Among female players, the RAE was found in the three most recent U17 tournaments, albeit weaker than the corresponding effect among male players. Additionally, the RAE was evident for the first time in the female U20 tournament in 2018. No (carryover) RAE was found in any senior female tournaments, which is probably due to the weaker effects in female U17 and U20 tournaments compared to boys; thus, the carryover effect did not extend to the senior women. These differences between sexes could be because of a higher number of players for selection in male soccer, and therefore fiercer competition among male players [56]. Thus, the more players that compete for a limited number of selection possibilities and the higher the competition level, the more likely the RAE will be evident [20].

In addition, since girls reach puberty earlier than boys, there is a possibility that the strictest selection among female players occurs at a time when the relative physical differences between players are smaller [71]. In the present data, effect sizes in female U17 tournaments are more similar to the male U20 tournaments (albeit still smaller) than to the male U17s, a shift of three years that could be at least partly explained by puberty-related differences. In addition, it could be argued that the physical differences between relatively early-born girls and their later-born peers might be less of a deciding factor than it is for boys, as women’s soccer is played slightly differently from men’s soccer due to the differences in game demands [72].

The general trend is that over time, the RAE has grown stronger for all groups of players (except senior women), as is indicated by the increase in effect sizes (see Fig 1). The most extreme effects are found in the more recent male U17 tournaments, in which almost three times as many players were born in Q1 compared to Q4. This indicates that the selection procedures are probably even more focused on present performance than on future prospects. Thus, the more physically developed boys will be picked over the slower developers, favoring the relatively earlier born [51].

Such bias is evident despite the fact that coaches are rather good at assessing players’ biological maturity age relative to their chronological age [73]. Despite this skill, these same coaches continue to systematically select those players who are more physically developed and tend to misconstrue physical development as skill [74]. When asked to evaluate players’ soccer-specific talent and giftedness, coaches associate more positive performance-related attributes with players of larger size [75]. This continues to happen more than thirty years after the RAE was discovered in sports, and after everyone involved in sports should be expected to be aware of the effect [67]. Indeed, coaches have been shown to be biased in the selection process even when they report being aware of the RAE [76]. The present data indicate that the selection procedures are seriously short-sighted.

The increase of the trend over time might also be because the game has in fact placed increasing physical demands on players, a trend that is also evident in youth soccer players who have been selected [67]. In addition, wages at the highest level have increased almost exponentially, making it ever more attractive to pursue a career as a soccer player [77, 78]. Moreover, top clubs have started recruiting increasingly younger players and have set up extensive scouting systems in their search for talent. It is reasonable to believe that such a trend would favor those players with initial physical advantages. As the pool of talented players seems to be increasing, it might be a tempting strategy to provide additional (e.g., Pygmalion [28] and Matthew [29]) effects to already more physically developed players instead of selecting players only based on skill. In any case, the present results indicate (as suggested by Helsen et al. [67]) that possessing knowledge about the existence of the RAE is not enough to avoid the effect.

In summary, the RAE is not always present, and when it is, it varies in strength. Although studies on the existence (or not) of the RAE across various domains are abundant, less is known about what exactly causes the differences in the observed results. Thus, predictions based on the theoretical tenets of the concept are difficult to confirm from the present literature. For example, it is difficult to establish how much of the effect stems from initial differences and how much is due to additional effects (most notably the Pygmalion effect and the Matthew effect). We would also suggest that soccer—by far the largest sport on the planet, with rather extreme selection mechanisms as well as bountiful rewards for those who persist to the highest level [77, 78]—lends itself nicely to such a study of the variations of the RAE.

In the context of soccer talent development, considerable consequences might be inferred from the potent RAEs reported in the present paper. Given that nothing indicates that soccer players born early in the year demonstrate overall better soccer performance compared to those born later in the same year [59, 79], the present findings give reason to believe that a serious loss of talent is experienced in the sport, due to excessive dropout rates among the relatively later born [45]. Furthermore, it would be safe to suppose that a talent selection and development system so heavily influenced by the RAE is probably wasting a lot of money on developing less-talented early-born players. Indeed, the RAE is even stronger among soccer players in the second tier [80], which might indicate that clubs below the top level are not in a financial position to compete for the special talents that have defied the RAE but must rather select from the larger pool of more affordable players who have advanced through the youth system via the more common route of being helped by the RAE [77, 78].

Among female soccer players, the competition between players has grown stronger in recent years, perhaps due to an almost exponential growth (nearly doubled from 2013 to 2017) in the number of professional players [81]. Furthermore, for the first time in the history of women’s soccer, it is possible for many more female players to pursue a career in soccer, as salaries are on the rise [82]. However, there are still many fewer girls playing soccer than boys, and the annual dropout rate among girls is much higher than among boys [83]; thus, the trend of an increasing RAE among girls is rather worrisome.

Even if much can be read and deduced from the multitude of published results on the RAE, studies included different groups with respect to age, sex, and playing level, and they also included different time frames (often showing mere snapshots, such as presenting the RAE among players within a single league over a single season). As a result, it remained uncertain how much the effect varies across different groups of players and how it has changed over time. Previous reviews on the topic, also, were unable to quantify the relative differences in the magnitude of the RAE across groups and over time. The present study includes directly comparable populations—at least as far as is practically possible—across players of different ages, both male and female, and over time; its findings demonstrate a trend of increasing RAEs in international soccer tournaments.

## Conclusions

The RAE is pervasive in world-championship-level soccer. The effect is strongest among male U-17 players and is similar, albeit somewhat weaker, among male U-20 players. Among male senior players, the RAE was present for the first time in 2014, and again in 2018. Among female players, the trend is similar to that among males, however weaker, and there is no carry-over RAE among senior female players. There is a trend of increasing strength of the RAE among all age-groups for both sexes. It is suggested that the trend may be due to increased selection pressure due to the increased financial rewards for players, and to increasing trading costs for clubs, that has led to scouting of players at younger ages.
